# Strut Deformation in CFRP-Strengthened Reinforced Concrete Deep Beams

**DOI:** 10.1155/2014/265879

**Published:** 2014-08-13

**Authors:** Mohammad Panjehpour, Hwa Kian Chai, Yen Lei Voo

**Affiliations:** ^1^Department of Civil Engineering, Faculty of Engineering, University of Malaya, 50603 Kuala Lumpur, Malaysia; ^2^DURA Technology Sdn Bhd, 31200 Chemor, Perak, Malaysia

## Abstract

Strut-and-tie model (STM) method evolved as one of the most useful designs for shear critical structures and discontinuity regions (D-regions). It provides widespread applications in the design of deep beams as recommended by many codes. The estimation of bottle-shaped strut dimensions, as a main constituent of STM, is essential in design calculations. The application of carbon fibre reinforced polymer (CFRP) as lightweight material with high tensile strength for strengthening D-regions is currently on the increase. However, the CFRP-strengthening of deep beam complicates the dimensions estimation of bottle-shaped strut. Therefore, this research aimed to investigate the effect of CFRP-strengthening on the deformation of RC strut in the design of deep beams. Two groups of specimens comprising six unstrengthened and six CFRP-strengthened RC deep beams with the shear span to the effective depth ratios (*a/d*) of 0.75, 1.00, 1.25, 1.50, 1.75, and 2.00 were constructed in this research. These beams were tested under four-point bending configuration. The deformation of struts was experimentally evaluated using the values of strain along and perpendicular to the strut centreline. The evaluation was made by the comparisons between unstrengthened and CFRP-strengthened struts regarding the widening and shortening. The key variables were *a/d* ratio and applied load level.

## 1. Introduction

Strut-and-tie model (STM) method evolved as one of the most useful designs for shear critical structures and discontinuity regions (D-regions). It reduces complex states of stress within the D-region in reinforced concrete (RC) member into a truss comprised of simple and uniaxial stress paths. The concrete stress field in strut, as a main constituent of STM, is usually wider at midlength of the strut than at the ends. Thus, strut generally varies in cross-section along its length. The strut that changes in its width along the length is idealised as a bottle-shaped geometry [1–7].

The cracking behaviour of bottle-shaped strut resembles that of concrete cylinder in a split tensile test. The internal lateral spread of applied compression force results in transverse tension strain in the strut which causes split cracking [2]. The bottle-shaped unreinforced strut longitudinally cracks while loading and it may cause ultimate failure of strut. Carbon fibre reinforced polymer (CFRP) has widespread applications for repair and strengthening of cracked concrete structural elements [8, 9]. The load capacity of strut can be increased by transverse reinforcement steel bar and external reinforcement of carbon fibre reinforced polymer (CFRP) sheet which restrain cracking. Dimensioning of strut, tie, and nodes is requisite to design the STM after computation of reactions and internal forces [10, 11]. Nevertheless, CFRP-strengthening of RC struts complicates the aforesaid dimensioning as CFRP-strengthening restrains the spread of stress fields.

STM method has been used for D-region analysis in structural elements such as deep beams according to many codes and standards [1–6]. Deep beams have been commonly used for high-rise buildings, offshore structures, and foundations [12], which are largely taken into account as a transfer girder in a single-span or continuous beam [7]. Experimental tests on reinforced concrete (RC) deep beams revealed that addition of web reinforcement beyond the recommended amount by design codes and standards was incapable of increasing the shear strength of deep beam [13]. Thus, CFRP sheet may be regarded as a proper material for external strengthening of D-regions in RC deep beams.

Prior research showed that the CFRP-strengthening increased the shear strength of RC deep beams in the range of 24–43% depending on the installation style and fibre direction of CFRP sheet [13]. This range was 35–73% for RC deep beams with the opening [14] and 10–66% for RC T-section deep beams [15]. The range of increase depended on the size of opening and CFRP-strengthening length, respectively. The strut effectiveness factor in STM recommended by ACI 318-05 was later modified for deep beam which was longitudinally reinforced by FRP bars with shear span to effective depth (*a/d*) ranging in 1.5 < *a*/*d* < 2.5 [16] and 1.4 < *a*/*d* < 2.1 [17]. To date, no research has been conducted on the effect of CFRP sheet bonding on RC strut behaviour, particularly the strut dimensions in STM of CFRP-strengthened D-region.

There are uncertainties regarding strut dimensioning and deformation while it is subject to load. Width of a strut has been estimated in the previous studies with the assumption that the strut has been stressed to the maximum [7, 18–21]. The dimensions estimation of bottle-shaped strut is essential in design calculations as the mechanical behaviour of strut is mainly influenced by its dimensions [2, 7, 19]. Among the latest publications on the STM and CFRP-strengthened RC deep beams [22–24] no research focused on the strut deformation subjected to load. Hence, determination of strut deformation under loading is vital for a reliable design. Furthermore, the strut deformation provides indication to the strut behaviour and subsequently to the overall behaviour of RC structural element.

The objective of this research is to investigate the effect of CFRP-strengthening on the deformation of RC strut in deep beam as it affects the failure of strut. The bonding between CFRP sheet and strut disrupts the load trajectories in strut and complicates the estimation of strut dimensions while loading. Hence, this investigation sets the scene for STM of CFRP-strengthened D-regions regarding strut dimensioning.

## 2. Methodology

Tests were performed on two groups of six RC deep beam specimens with and without CFRP-strengthening. Each group of beams had the shear span to the effective depth ratios of 0.75, 1.00, 1.25, 1.50, 1.75, and 2.00. Both groups of beam specimens were identical in every aspect, excluding the condition of strengthening by CFRP sheets. The variables of the investigation were* a/d *ratio and the applied load level as these two variables affect the deformation of struts and mechanical behaviour of RC deep beams.

**Figure 1:**
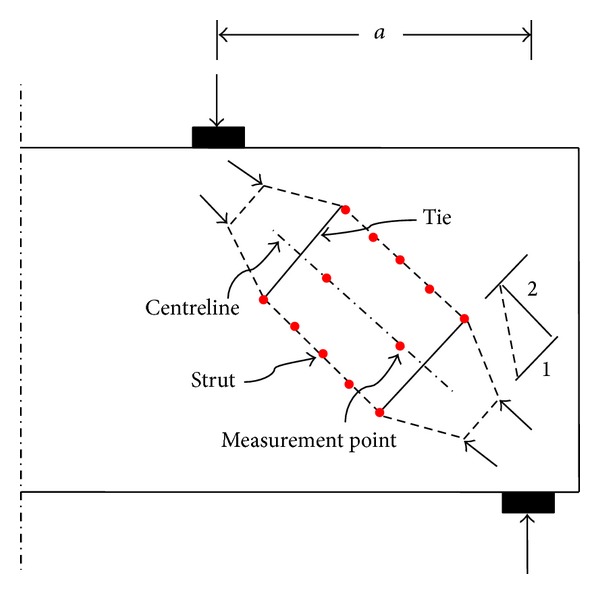
Strut-and-tie model of a bottle-shaped strut including measurement points.

A typical bottle-shaped strut is illustrated in Figure 1. Bulging stress trajectories generates considerable transverse stress in the midlength of strut with compression in the neck and tension further away leading to longitudinal cracking. The dashed outline in Figure 1 indicates approximately the boundaries of bottle-shaped strut. The bottle-shaped strut consists of other struts and ties as shown in Figure 1 using dashed line and solid line, respectively [2].

The CFRP sheet was utilised in the current experiment as an external reinforcement to restrain cracking of RC struts in deep beams. The outline of bottle-shaped strut shown in Figure 1 is mainly based on the load path from support plate to load plate. The deformation of strut in unstrengthened and CFRP-strengthened D-region was evaluated by measuring the average values of strains along and perpendicular to the strut centreline. A demountable mechanical strain gauge (DEMEC) was utilised for strain measurement. The shear failure of deep beams was dominant in this experiment. The shortening and widening of the strut were measured in each step of loading with an increment of 50 kN till failure of strut.

### 2.1. Deep Beams Details

**Figure 2:**
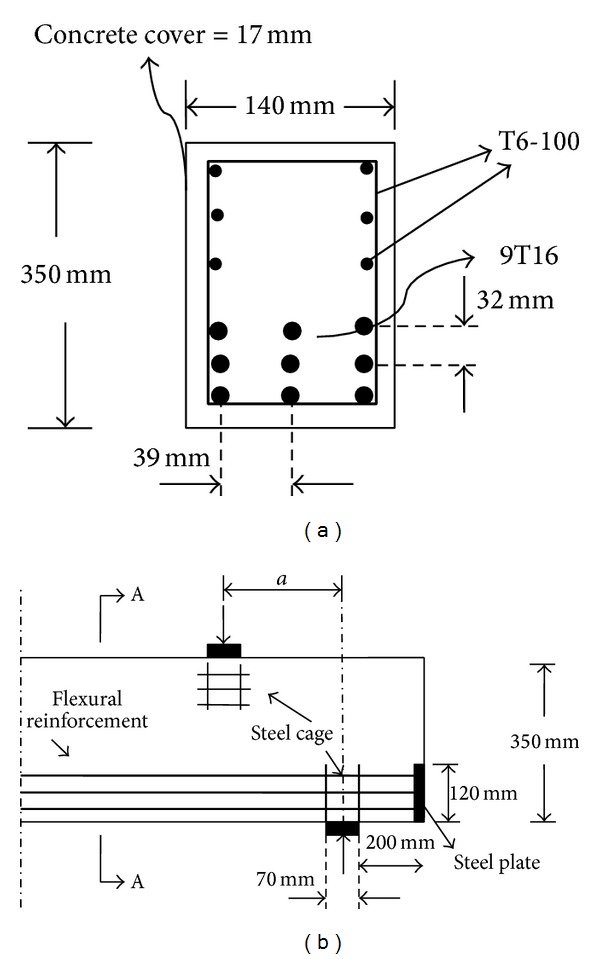
Beam details for half length: (a) section A-A, (b) side view.

All the beam specimens were 1840 mm in length, 140 mm in width, and 350 mm in height. Nine deformed steel bars of 16 mm diameter were longitudinally placed in three layers in the bottom of beams as flexural reinforcement. Orthogonal grids of reinforcement with the spacing of 100 mm were provided at two sides of beams using deformed steel bars of 6 mm diameter. The longitudinal steel bars were welded to the two end steel plates to provide adequate anchorage. The end steel plates with 120 mm of height and 10 mm of thickness covered the width of beams fully at both ends of beams. The additional reinforcements (steel cage) using deformed steel bars of 6 mm diameter were placed under the load plates and atop the support plates to prevent local bearing stress. The beams details are indicated in Figure 2.

### 2.2. Material and Specimens Preparation

**Figure 3:**
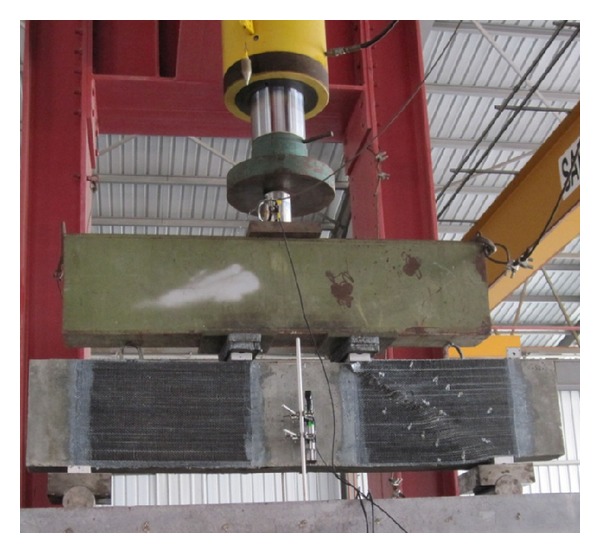
Test setup.

Ordinary Portland concrete with 28-day cylindrical compressive strength of 37.02 MPa and splitting tensile strength of 3.31 MPa was used to fabricate all the beams. The maximum aggregate size and water to cement ratio were 10 mm and 0.48 in the concrete mix design, respectively. The density of concrete was 2420 kg/m^3^. One layer of unidirectional CFRP sheet with the thickness of 0.111 mm/ply and two-part epoxy impregnation resin were utilised for strengthening. The rectangular surface areas between load and support plates (D-region) were fully covered by CFRP sheet at two sides of beams using wet lay-up system as shown in Figure 3.  Table 1 presents the typical properties of the CFRP sheet and epoxy resin provided by the manufacturer. The CFRP sheet and epoxy resin were supplied by Sika Company with product data sheet of sikawrap-230C and sikadur-330, respectively. The required curing time for CFRP sheet after bonding was two days at ambient temperature as recommended by the manufacturer. The support plates and the load plates, which were 70 mm in width and 10 mm in thickness, fully covered the bottom and top of the beams. All the beams were cured for two weeks after casting with a wet mat.

### 2.3. Instrumentation and Test Procedure

All the beams were simply supported over the two steel plates and tested under four-point bending configuration. The uniformly increasing load was applied with an increment of 50 kN using a hydraulic actuator with a maximum capacity of  5000 kN. The surface of concrete was carefully rubbed with sand paper before bonding of CFRP sheets. To prepare two-part epoxy resin based on the manufacturer recommendations, the hardener and resin were mixed and stirred for three minutes with a proportion of one to four. After two-week curing of the beams, the CFRP sheets were installed and covered the shear span of beams at two sides.

The DEMEC discs were installed along and perpendicular to the strut centreline in all the beams with a distance of 200 mm equal to the length of DEMEC bar as shown in Figure 1. The resolution of DEMEC gauge was 0.001 mm. In an attempt to obtain the accurate average values of tensile strains for evaluating strut widening, the transverse tensile strains were measured in five positions with the same distance, perpendicular to the strut centreline as shown in Figure 1. The value of compressive strains was also measured along the strut centreline. The deep beams were tested at 28 days after casting.

## 3. Experimental Results and Discussion

Prior to cracking, the elastic stress field occurred in the D-region of RC deep beams, which can be quantified with elastic analysis. Cracking occurred in the D-region as the applied load increased and disrupted the stress field which led to disorientation of the internal load path. The STM method rationally embodies a system of forces which is in equilibrium with a given set of loads. However, CFRP-strengthening disrupted the load trajectories in strut and complicated the disorientation of internal forces. Therefore, the estimation of CFRP-strengthened strut dimensions under loading became inaccurate.

### 3.1. Ultimate Shear Strength and Failure Mode of Beams

**Figure 4:**
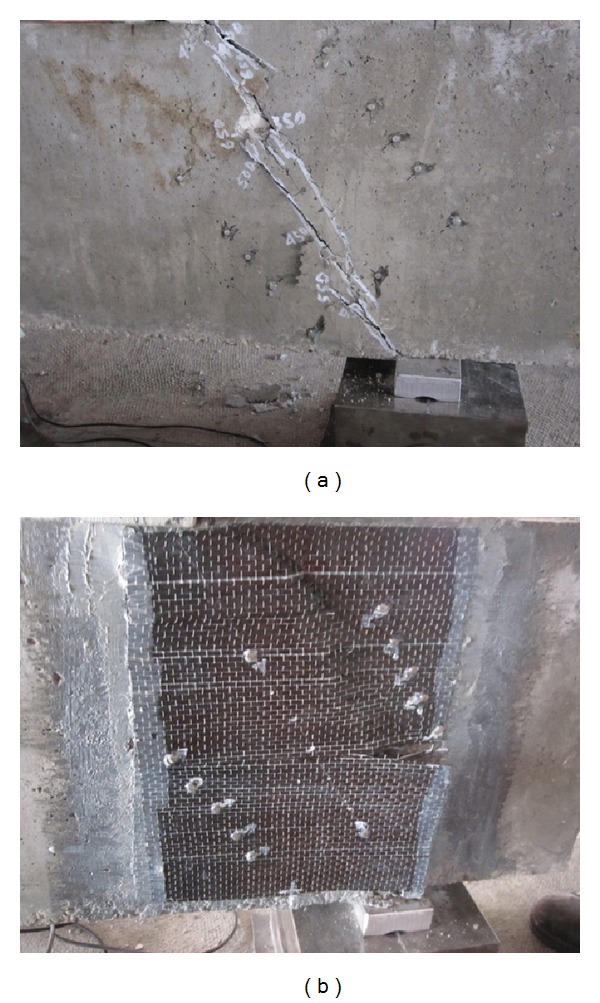
Typical failure of strut in RC deep beams with *a*/*d* = 0.75: (a) ordinary RC deep beam, (b) CFRP-strengthened RC deep beam.

In this experiment, shear failure mode with diagonal crack propagating towards the load plate and support plate was dominant for unstrengthened RC deep beams as expected based on the prior research [12]. The application of steel cage under the loading plates and atop the support plates was successful as no bearing failure was observed in these areas. The CFRP-strengthening significantly increased the ultimate shear strength of RC deep beams as shown in Table 2. The rupture of CFRP sheets was dominant in shear failure of all CFRP-strengthened RC deep beams. Typical failure mode of unstrengthened and CFRP-strengthened RC struts in deep beams from the experiment is shown in Figure 4.

### 3.2. Strut Dimensioning

The orientation of strut is affected by the ratio of* a/d*. In addition, the behaviour of strut changes from elastic to plastic with the increase of applied load level. Therefore, the two variables of* a/d* ratio and applied load level were chosen for the evaluation. The experimental results showed that the strut deformation in the transverse direction was greater than the direction along the strut centreline for both the ordinary and the CFRP-strengthened specimens. This was because the strut tended to widen more than shorten due to the cracking occurring parallel to the strut centreline which caused greater values of transverse strains than compressive strains. Moreover, the effect of CFRP-strengthening on the strut compressive strain and consequently the strut shortening was insignificant.

#### 3.2.1. Strut Widening

The cracking and crack widening caused softening behaviour in RC strut which affected strut dimensioning and consequently the strut behaviour under loading. The CFRP-strengthening of RC strut restricted the strut widening compared to the unstrengthened status.

**Figure 5:**
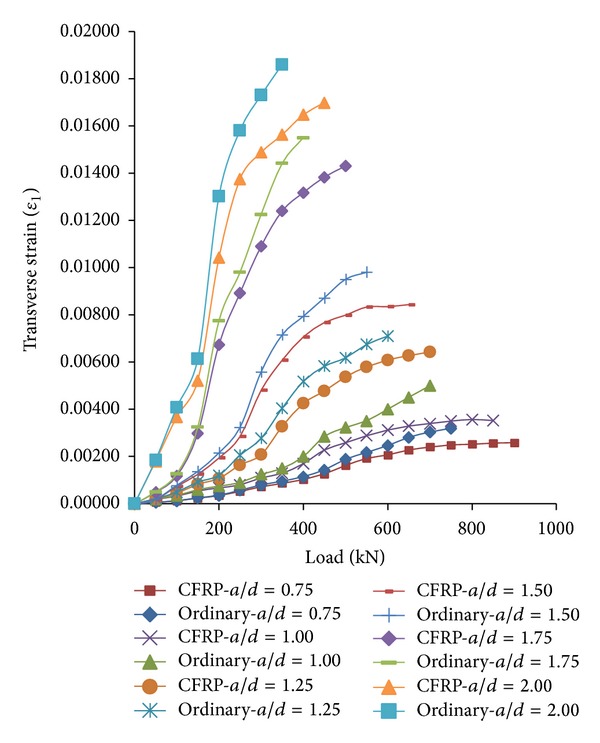
Load-transverse strain curves of unstrengthened and CFRP-strengthened RC struts.

*(1) General Investigation on RC Strut Widening.* The load-transverse strain curves of the ordinary and the CFRP-strengthened RC struts from the experiment are shown in Figure 5. The figure demonstrates that the value of strut transverse strain for both the ordinary and the CFRP-strengthened RC struts increased with increasing of applied load. In addition, for the same applied load, the transverse strain and strut widening of the specimens with higher* a/d* were found to be greater than those with lower* a/d*. The difference between the transverse tensile strains of the ordinary and the CFRP-strengthened RC struts (Δ*ɛ*) with load step of 100 kN is presented in Table 3.

Based on Table 3, the increase of* a/d* ratio resulted in increase of  Δ*ɛ*. Therefore, with the same applied load, the strut widening in the ordinary strut increased more than in the CFRP-strengthened strut as* a/d* ratio increased. It implied that the effect of CFRP-strengthening on restraining crack widening increased with the increase of* a/d* ratio. This was because the diagonal cracks were propagated in a larger area for high* a/d* ratio than those of low* a/d* ratio which resulted in the stress field distribution in a wider strut. Therefore, the stress field was transferred to different random portions of CFRP sheet through more diagonal cracks in high* a/d* beams compared to those of low* a/d*.

Based on Table 3, for the same* a/d* ratio, the increase of applied load resulted in greater value of Δ*ɛ*. Therefore, for the same* a/d*, the strut widening increased in the ordinary strut more than those of CFRP-strengthened strut as the applied load increased. This was due to the increasing crack widening in the high applied load compared to those of low applied loads. Thus, the effect of CFRP-strengthening appeared to be more apparent in higher applied load level.

Figure 5 illustrates the load-transverse strain curve of struts which consisted of two main parts for both groups of ordinary and CFRP-strengthened RC deep beams. The former was approximately a line extended up to about 40–60% of ultimate shear strength of beams. The linear trend of transverse strain-load curve was nearly similar for both ordinary and CFRP-strengthened struts. Thus, the effect of CFRP-strengthening on the strut widening was insignificant in this part of the curve due to the slight crack widening and CFRP sheet was not remarkably involved to restrain the crack widening. The latter was approximately a part of parabolic curve with extrinsic curvature as shown in Figure 5. The extrinsic curvatures part of the curves for CFRP-strengthened and ordinary RC struts became open and dissimilar from each other with increasing applied load level as opposed to the first linear part of curves. It was due to the fact that plastic behaviour of concrete was dominant in this part of curves instead of elastic behaviour which occurred in the linear part. Thus, the CFRP sheet was involved to restrain the crack widening, crushing of reinforced concrete and consequently transverse strains in CFRP-strengthened struts. Therefore, the amounts of transverse strains for ordinary strut remarkably grew faster than those of CFRP-strengthened struts, and it caused the parabolic parts of ordinary and CFRP-strengthened RC strut to go further away from each other.

Based on the experimental results, the value of transverse strain of ordinary RC struts remarkably grew faster than that of CFRP-strengthened RC struts after the amount of loading was higher than approximately 40–60% of ultimate shear strength of ordinary RC deep beams. Hence, the strut widening was remarkably affected by CFRP-strengthening after the applied load was higher than approximately 40–60% of ultimate shear strength of ordinary RC deep beams. The amount of Δ*ɛ* was negligible in the linear part of curves which was due to minimal crack widening. Therefore, the effect of CFRP on the strut widening was insignificant in the low applied load level.

*(2) Strut Widening with Different Applied Load Levels.* The average transverse strain of struts for both ordinary and CFRP-strengthened RC deep beams was classified in two groups according to applied load level. The strain-load curves were very close in distance and similar in pattern for the amounts of applied load lower than 50% of ultimate load as shown in Figure 5. Therefore, the applied load greater than 50% of ultimate load was taken into consideration in this section of the research as the strain-load curves were distinctly separated from each other in this level of loading.

**Figure 6:**
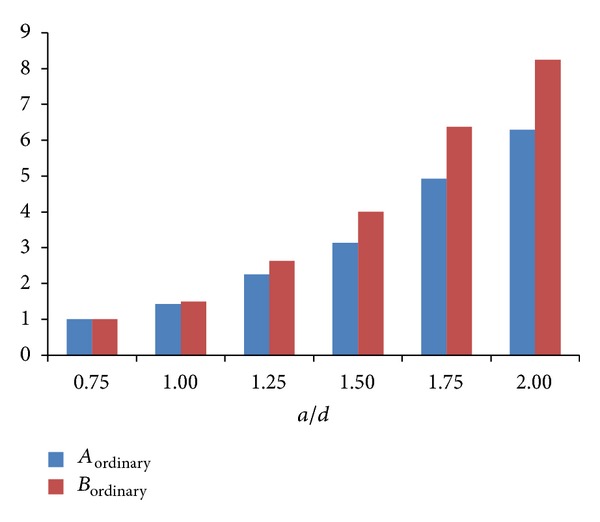
Comparison of average transverse strains of unstrengthened RC struts for different applied load levels.

The value of *ɛ*_50−75_ represents the average of transverse strain corresponding to 50% and 75% of ultimate shear load. The same classification was used for 75% and 100% of ultimate shear load regarding the value of *ɛ*_75−100_. The values of *ɛ*_50−75_ and *ɛ*_75−100_ for deep beams with *a*/*d* = 0.75 were assumed as a basis for normalisation as shown in Tables 4 and 5. The normalised values in Tables 4 and 5 depicted that, with the increase of* a/d*, the average transverse strain for ordinary and CFRP-strengthened struts rose to approximately 8 and 7 times compared to those of struts with *a*/*d* = 0.75, respectively. This increase followed approximately the same trend for both applied load classifications of 50–75% and 75–100% in ordinary RC deep beams. Based on Figure 6, the value of *B*_ordinary_ grew faster than that of *A*_ordinary_ as* a/d* increased. It implied that with the increase of* a/d* the strut widening and the increase rate of widening for load classification of 75–100% was greater than that of 50–75%. This was because the propagated cracks occurred in range of 50–75% applied load and then developed and widened when applied load reached the range of 75–100%. Thus, it resulted in greater widening of strut.

**Figure 7:**
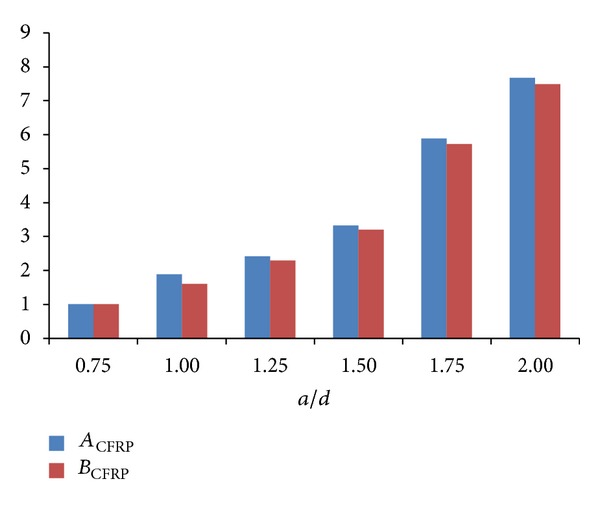
Comparison of average transverse strains of CFRP-strengthened RC struts for different applied load levels.

The values of *A*_CFRP_ and *B*_CFRP_, which were indicated in Figure 7, increased as* a/d* ratio increased. However, the rate of increase of *B*_CFRP_ was lower than that of *A*_CFRP_ as opposed to the ordinary strut as shown in Figure 7. Therefore, the increase rate of widening of CFRP-strengthened strut in load classification of 75–100% was lower than that of 50–75% as opposed to the ordinary struts. While beam was under the load classification of 50–75%, the CFRP sheet was stretched. Due to the restriction of crack widening by CFRP sheet, the strut widening was lower than the ordinary status. The aforesaid restriction by CFRP sheet was greater while beam was under 75–100% load classification because the CFRP sheet sustained higher tensile stress which was closer to its ultimate tensile stress compared to the ordinary status. The difference of *A*_CFRP_ and *B*_CFRP_ became less apparent as* a/d* ratio increased as shown in Figure 7. This effect was due to the applied load for high values of* a/d* ratio which were proportionally less than low values of* a/d* ratio.

#### 3.2.2. Shortening of RC Strut

**Figure 8:**
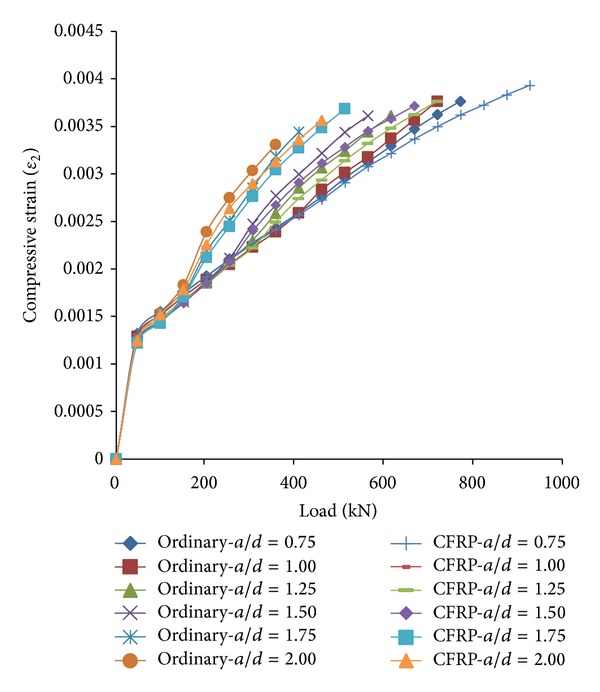
Load-compressive strain curve of unstrengthened and CFRP-strengthened RC struts.

The load-compressive strain curves consisted of two parts for both ordinary and CFRP-strengthened RC deep beams as shown in Figure 8. The former was a linear part extending from the beginning to the approximate amounts of compressive strains which was in the range of 0.0010 to 0.0015. The direction of linear part then turned downward sloping into a parabolic curve with an extrinsic curvature lower than the same curve for load-transverse strain. As opposed to the load-transverse strain, the curves of load-compressive strains for the ordinary and the CFRP-strengthened RC struts were close enough to appear almost similar which can be hardly differentiated as shown in Figure 8. However, the shortening amount for both of the ordinary and the CFRP-strengthened RC strut was not comparable with those of widening amounts. This was due to the deformation of the bottle-shaped strut which was mainly governed by the transverse stresses acting perpendicular to the strut centreline which significantly affected the widening of struts.

The effect of CFRP-strengthening on the RC strut shortening was negligible. The curves in Figure 8 indicated that the value of compressive strain for CFRP-strengthened RC strut was lower than that of the ordinary strut. CFRP sheet mechanical properties were conclusively not the cause of compressive strain reduction as this material is unable to sustain compressive stress. The increase of strut widening resulted in increase of strut shortening, and vice versa. The restraint of strut shortening was due to strut widening restrained by CFRP sheet. Therefore, the shortening of CFRP-strengthened RC struts was lower than that of ordinary struts.

## 4. Conclusion

Tests were performed on two sets of six RC deep beam specimens with and without CFRP-strengthening. The objective of the research was to investigate the RC strut deformation regarding its widening and shortening in the design of deep beams. The key variables were shear span to effective depth ratio and applied load level.

In deep beams subjected to load, as the level of widening of strut increases, the compressive strength of strut along its longitudinal axis decreases. Consequently the ultimate shear strength of deep beam decreases. Thus, designer of deep beam must pay attention to the significant effects of* a/d* ratio and applied load level on the widening of strut subjected to load. Nevertheless, the CFRP-strengthening of strut reduces the strut widening and increases the ultimate shear strength of deep beams. The experimental results regarding the effects of* a/d* ratio and applied load level on the strut widening for design purposes of deep beams with and without CFRP-strengthening are given below. The conclusion is drawn in terms of general aspects (items 1 and 2), similar widening behaviour (items 3, 4, and 9), distinctive widening behaviour (items 5, 6, 7, 8, 10, and 11), and shortening behaviour (item 12) of RC strut with and without CFRP-strengthening.

1. The CFRP-strengthening significantly increased the shear strength of RC deep beams.
2. Strut deformation in the transverse direction was greater than the direction along the strut centreline for both conditions of with and without CFRP-strengthening.
3. As the applied load increased the values of transverse strain in both the ordinary and the CFRP-strengthened RC struts increased.
4. With the same applied load, the transverse strain, and consequently the strut widening in both the ordinary and the CFRP-strengthened RC deep beams with high* a/d* was greater than those of low* a/d*.
5. With the same applied load, strut widening in ordinary strut increased more than CFRP-strengthened strut as the* a/d* increased.
6. The effect of CFRP-strengthening on crack widening restraint increased with the increase of* a/d* ratio. Thus, CFRP sheet contributed to strengthening of RC struts in high* a/d* ratio more than in low* a/d* ratio.
7. With the same value of* a/d*, the strut widening of ordinary strut increased more than those of CFRP-strengthened strut as the applied load increased. Thus, the effect of CFRP-strengthening on restriction of strut widening appeared to be more apparent in higher applied load.
8. The value of transverse strain of ordinary RC struts remarkably grew faster than that of CFRP-strengthened RC struts after the amount of loading was higher than approximately 40–60% of ultimate shear strength of ordinary RC deep beams. Therefore, the strut widening was significantly affected by CFRP-strengthening after approximately 40–60% of ultimate shear strength of ordinary RC deep beams.
9. With the increase of* a/d* ratio up to 2.00, the average transverse strain of the ordinary and the CFRP-strengthened struts increased up to approximately 8 and 7 times compared to that of strut with *a*/*d* = 0.75, respectively.
10. With the increase of* a/d*, widening of ordinary RC strut increased, and the increase rate of widening for load classification of 75–100% was greater than that of 50–75%.
11. With the increase of* a/d*, widening of CFRP-strengthened RC strut increased. However, the increase rate of widening of CFRP-strengthened struts in load classification of 75–100% was lower than that of 50–75% as opposed to the ordinary struts.
12. The increase of strut widening resulted in the increase of strut shortening, and vice versa. The strut shortening was restrained as the CFRP sheet restrained the strut widening. Therefore, the shortening of CFRP-strengthened RC struts was lower than that of ordinary struts.

## Supplementary Material

The Tables 1 and 2 represent the average value of transverse strain of ordinary and CFRP-strengthened RC struts from experiment respectively.

## Figures and Tables

**Table 1:** Typical properties of CFRP sheets and epoxy.

Materials	Tensile strength (MPa)	Tensile modulus of elasticity (GPa)	Bond strength (MPa)	Fabric thickness (mm/ply)	Laminate thickness impregnated with resin (mm/ply)
CFRP sheet	3900	230	—	0.111	0.9
Epoxy resin	30	4.5	>4	—	—

**Table 2:** Ultimate shear strength of RC deep beams.

*a*/*d*	*P*_u-ordinary_ (kN)	*P*_u-CFRP-strengthening_ (kN)
0.75	756.95	905.31
1.00	709.01	857.89
1.25	604.08	740.02
1.50	555.91	691.04
1.75	403.02	510.01
2.00	360.02	468.05

**Table 3:** Difference of transverse tensile strains of the unstrengthened and CFRP-strengthened RC struts.

	Load (kN)							
		100	200	300	400	500	600	700
*a*/*d*	0.75	0.0000	(0.0000)	0.0001	0.0001	0.0003	0.0004	0.0006
	1.00	0.0000	(0.0001)	0.0002	0.0003	0.0006	0.0009	0.0018
	1.25	0.0000	(0.0001)	0.0007	0.0009	0.0008	0.0010	—
	1.50	0.0000	(0.0002)	0.0008	0.0009	0.0015	—	—
	1.75	0.0000	(0.0010)	0.0013	0.0023	—	—	—
	2.00	0.0004	(0.0026)	0.0024	—	—	—	—

**Table 4:** Average transverse strains of unstrengthened RC struts for different applied load levels.

Number	*a*/*d*	ɛ_50–75_	ɛ_75–100_	*A*_ordinary_	*B*_ordinary_
1	0.75	0.0028	0.0032	1.00	1.00
2	1.00	0.0040	0.0048	1.43	1.50
3	1.25	0.0063	0.0084	2.25	2.63
4	1.50	0.0088	0.0128	3.14	4.00
5	1.75	0.0138	0.0204	4.93	6.38
6	2.00	0.0176	0.0264	6.29	8.25

**Table 5:** Average transverse strains of CFRP-strengthened RC struts for different applied load levels.

No	*a*/*d*	*ɛ*_50–75_	*ɛ*_75–100_	*A*_CFRP_	*B*_CFRP_
1	0.75	0.0018	0.0025	1.00	1.00
2	1.00	0.0034	0.0040	1.89	1.60
3	1.25	0.0044	0.0058	2.42	2.30
4	1.50	0.0060	0.0080	3.33	3.20
5	1.75	0.0106	0.0143	5.89	5.72
6	2.00	0.0138	0.0187	7.67	7.48

## Symbols

*ɛ*_50−75_: The average of transverse strain corresponding to 50% and 75% of ultimate shear load
*ɛ*_75−100_: The average of transverse strain corresponding to 75% and 100% of ultimate shear load
*ɛ*_ordinary_: The average of transverse tensile strain of ordinary unstrengthened RC strut
*ɛ*_CFRP_: The average of transverse tensile strain of CFRP-strengthened RC strut
Δ*ɛ* = *ɛ*_ordinary_ − *ɛ*_CFRP_: The difference value between the transverse tensile strains of ordinary unstrengthened and CFRP-strengthened RC strut
*A*_ordinary_: The ratio of *ɛ*_50−75_ for each ordinary unstrengthened beam with* a/d* ranging in 1.00–2.00 to *ɛ*_50−75_ for beam with *a*/*d* = 0.75
*B*_ordinary_: The ratio of *ɛ*_75−100_ for each ordinary unstrengthened beam with* a/d* ranging in 1.00–2.00 to *ɛ*_75−100_ for beam with *a*/*d* = 0.75
*A*_CFRP_: The ratio of *ɛ*_50−75_ for each CFRP-strengthened beam with* a/d* ranging in 1.00–2.00 to *ɛ*_50−75_ for beam with *a*/*d* = 0.75
*B*_CFRP_: The ratio of *ɛ*_75−100_ for each CFRP-strengthened beam with* a/d* ranging in 1.00–2.00 to *ɛ*_75−100_ for beam with *a*/*d* = 0.75
*a/d*: Shear span to effective depth ratio
*P*_u-ordinary_: Ultimate shear strength of ordinary unstrengthened RC deep beam from the experiment (kN)
*P*_u-CFRP-strengthening_: Ultimate shear strength of CFRP-strengthened RC deep beam from the experiment (kN).
