# Supplementary material for: Strut Deformation in CFRP-Strengthened Reinforced Concrete Deep Beams
Source: ScientificWorldJournal. 2014 Aug 13;2014:265879. doi: 10.1155/2014/265879 (PMC4147263; doi:10.1155/2014/265879)
Supplement: Supplementary file 1 — The Tables 1 and 2 represent the average value of transverse strain of ordinary and CFRP-strengthened RC struts from experiment respectively. [file 265879.f1.pdf]

Appendix

The Tables 1 and 2 represent the average value of transverse strain of ordinary and CFRP-strengthened RC struts from experiment respectively.

Table 1. Average transverse strain of ordinary unstrengthened RC struts

	$\varepsilon_{1\text{-ordinary}}$					
**Load (kN)-CFRP**	**a/d=0.75**	**a/d=1.00**	**a/d=1.25**	**a/d=1.50**	**a/d=1.75**	**a/d=2.00**
0.00	---	---	---	---	---	---
50.00	0.0001	0.0002	0.0002	0.0002	0.0005	0.0019
100.00	0.0001	0.0004	0.0005	0.0008	0.0013	0.0041
150.00	0.0003	0.0006	0.0009	0.0014	0.0033	0.0061
200.00	0.0004	0.0008	0.0012	0.0022	0.0077	0.0130
250.00	0.0006	0.0009	0.0021	0.0032	0.0098	0.0158
300.00	0.0008	0.0013	0.0028	0.0056	0.0123	0.0173
350.00	0.0010	0.0015	0.0040	0.0072	0.0144	0.0186
400.00	0.0012	0.0020	0.0052	0.0079	0.0155	---
450.00	0.0014	0.0029	0.0058	0.0087	---	---
500.00	0.0019	0.0032	0.0062	0.0095	---	---
550.00	0.0022	0.0035	0.0067	0.0098	---	---
600.00	0.0025	0.0040	0.0071	---	---	---
650.00	0.0028	0.0045	---	---	---	---
700.00	0.0030	0.0050	---	---	---	---
750.00	0.0032	---	---	---	---	---

Table 2. Average transverse strain of CFRP-strengthened RC struts

	$\varepsilon_{1\text{-CFRP-strenghtnend}}$					
**Load (kN)-CFRP**	**a/d=0.75**	**a/d=1.00**	**a/d=1.25**	**a/d=1.50**	**a/d=1.75**	**a/d=2.00**
0.00	0.0000	0.0000	0.0000	0.0000	0.0000	0.0000
50.00	0.0001	0.0002	0.0002	0.0002	0.0005	0.0018
100.00	0.0001	0.0003	0.0004	0.0007	0.0012	0.0037
150.00	0.0002	0.0005	0.0008	0.0012	0.0030	0.0052
200.00	0.0003	0.0007	0.0011	0.0019	0.0067	0.0104
250.00	0.0005	0.0008	0.0016	0.0028	0.0089	0.0138
300.00	0.0007	0.0011	0.0021	0.0048	0.0109	0.0149
350.00	0.0009	0.0013	0.0033	0.0061	0.0124	0.0156
400.00	0.0010	0.0017	0.0043	0.0071	0.0132	0.0165
450.00	0.0013	0.0023	0.0048	0.0077	0.0138	0.0170
500.00	0.0016	0.0026	0.0054	0.0080	0.0143	
550.00	0.0019	0.0029	0.0058	0.0083	---	---
600.00	0.0021	0.0031	0.0061	0.0083	---	---
650.00	0.0023	0.0032	0.0063	0.0084	---	---
700.00	0.0024	0.0033	0.0064	---	---	---
750.00	0.0025	0.0033	---	---	---	---
800.00	0.0025	0.0035	---	---	---	---
850.00	0.0026	0.0035	---	---	---	---
900.0000	0.0026	---	---	---	---	---
